# Plant lectins and Toll-like receptors: implications for therapy of microbial infections

**DOI:** 10.3389/fmicb.2014.00020

**Published:** 2014-02-03

**Authors:** Luís C. N. da Silva, Maria T. S. Correia

**Affiliations:** Departamento de Bioquímica, Universidade Federal de PernambucoRecife, Brazil

**Keywords:** TLR receptors, immunomodulation, lectin, microorganisms, infection

Several plant lectins are known as potent immunomodulatory agents, and they are applied in different experimental models of infection (Afonso-Cardoso et al., 2011; Oliveira et al., 2013). Recent research has characterized these proteins as potential agonists of Toll-like receptors (TLRs or TLR), a family of mammalian homologs of *Drosophila* Toll protein involved in the detection of microbes and initiating inflammatory responses (O'Neill et al., 2013). In this opinion article, we highlighted some studies of plant lectins as modulators or agonists of TLRs in order to stimulate research interest in this fascinating area and promote knowledge sharing and scientific collaboration.

## A short view on plant lectins

Lectins are a very large class of carbohydrate-binding proteins of non-immune origin. Through the interaction with sugars, they trigger several important cellular processes. Lectins are universally expressed and have been shown to function in animals, plants, and microorganisms as cell and molecular recognition proteins. However, the carbohydrate-binding domains have been studied most intensively within the plant kingdom (Sharon, 2007; Vandenborre et al., 2011).

The discovery of plant lectins occurred in the 19th century, however, many questions about the biological role of these molecules remain obscure. Lectins may be involved in sugar transport, carbohydrate storage and they are associated as molecular chaperones (Van Damme et al., 2004; Liu and Li, 2013). The adhesion and agglutination properties of lectins have been related in the interaction of both symbiotic and pathogenic interaction of some microorganisms and host (Audfray et al., 2012). Plant lectins represent a group of proteins with obvious differences in their biochemical/physicochemical properties, molecular structure, carbohydrate-binding specificity and biological activities (Liu et al., 2010). Lectins are oligomeric which exhibit a large structural diversity and the molecular size range from 60 to 400 kDa. Each lectin polypeptide contains many molecular domains, one of which is the non-catalytic carbohydrate recognition domain, responsible for their ability to recognize and interact with specific glycoconjugates, without altering their structure. In the past few years, hundreds of plant lectins have been purified and characterized in details with respect to their biochemical properties, carbohydrate-binding specificities, these approaches allowed their classification (Van Dammes et al., 2011).

## Toll-like receptors

Plant materials represent an excellent source of immune modulators, which have been appointed as a new approach for combating infections caused by resistant microorganisms (Hancock et al., 2012). In this sense, a range of potential compounds have been proposed as agonists or inductors of immune receptors, including TLRs.

The TLR family is the best characterized group of innate immune receptors in terms of known ligands, downstream signaling pathways and functional relevance. They comprise a family of receptors homologous to Toll receptor from Drosophila melanogaster. In humans, the TLR family includes 10 transmembrane proteins that play a crucial role in host defense: they recognize molecular characteristics of microorganisms, known as pathogen-associated molecular patterns (PAMPs) highly conserved between different classes of microorganisms. For example, TLR4 and accessory proteins recognize lipopolysaccharide (LPS), while TLR2 recognizes lipoteichoic acid and various lipopeptides (when in complex with either TLR-1 or TLR-6), and TLR5 recognizes flagellin (Hancock et al., 2012; O'Neill et al., 2013). In this way, TLR receptors represent important therapeutic targets for developing new drugs able to directly modulate the host response against microbial infection. In fact, some TLR agonists and TLR modulators have been investigated as potential drugs on clinical trials and research programmes (Hennessy et al., 2010; Murgueitio et al., 2012).

## Plant lectins and toll-like receptors

Lectins have been extensively used as valuable tools in the biomedical research. The versatility of these biomolecules is due to their interactions with receptor-linked glycans on cell surfaces, which may trigger cell signaling and physiological responses (Lam and Ng, 2011). Plant lectins are characterized as immunomodulator agent, which result in the production of certain cytokines and reactive species and induce efficient immune responses against tumors or microbial infections. A very comprehensive review of immunomodulatory lectins has recently been published by Souza et al. (2013). In order to investigate the mechanism of this action, some researchers correlated the activation of immune cells by lectins with the expression of TLR receptors.

Sodhi et al. (2007) investigated the expression of different TLRs induced by the famous lectin from Concanavalin A (Con A) using mouse macrophages as a model. Con A enhanced *in vitro* expression of TLRs (2–9) and its action was related with JNK, p38, p42/44, and NF-κ B. The authors also showed the heterodimerization of TLR-2 and TLR-6. Additionally, Con A pre-treated-macrophages were more susceptible to induction of proinflammatory cytokines and nitric oxide by different TLR ligands (ZymosanA, PolyI:C, LPS, CpG DNA).

In other study, the Korean mistletoe lectin (KML-C) from *Viscum album coloratum* was shown to be a potent activator of TLR-4. The treatment of mouse peritoneal macrophages with KML-C-induced the upregulation of interleukin-1 receptor-associated kinase-1 (IRAK1) resulting in macrophage activation and TNF-α production, which was not observed when TLR-4 was blocked using a TLR-4-specific neutralizing antibody or TLR-4-deficient macrophages. The expression of TLR-4 was also induced by lectin-like protein from *Anoectochilus formosanus* (IPAF), resulting in the stimulation of TNF-α and IL-1β, CD86, and MHC II and phagocytic activity (Park et al., 2010).

The capacity to modulate the TLR were explored to combat the experimental infection of *Paracoccidiodes brasiliensis* using native and recombinant KM^+^, a mannose-binding lectin from *Artocarpus integrifolia*. BALB/c mice were infected with *P. brasiliensis* and after 10 days both proteins were separately administered. KM^+^ treatment reduced significantly colony-forming unit and induced higher levels of nitric oxide, INF-α, TNF-α, and IL-12, which was dependent on TLR-2 (Coltri et al., 2008).

Recently, in a remarkable paper, phytohaemagglutinin (PHA from *Phaseolus vulgaris*) and its isoforms were showed as a specific human TLR-4 agonist during an initial screening. This result encouraged the authors to examine the effects of this and other lectins on external (-2/6, -4, and -5) and internal (-3, -7, -8, and -9) human TLRs. In this research, SBA (Soybean agglutinin from *Glycine max*), PNA (peanut agglutinin from *Arachis hypogaea*), ConA and PHA only stimulated extracellular TLRs (-2/6, -4, or -5): TLR-4 for SBA and PNA; TLR-2/6 for ConA; TLR-2/6, -4 and for PHA-L. In other hand, WGA (wheat germ agglutinin from *Triticum vulgaris*) was the most promiscuous lectin activating all tested receptors, except TLR-3 and -4. The jacalin (from *Artocarpus integrifolia*) was inactive. This variety of TLR agonist pharmacology is related to different sugar ligand specificity of each plant lectins, suggesting that the action is encoded by the carbohydrate recognition motifs on different TLRs (Unitt and Hornigold, 2011). TLR agonists have been proposed as adjuvants for vaccines against virus (Behzad et al., 2012; Hong et al., 2012), bacteria (Hancock et al., 2012), parasite (Moon et al., 2012) and fungi (LeibundGut-Landmann et al., 2012).

In conclusion, these observations encourage further studies for the characterization of plant lectins as novel agonists and modulators of TLR receptors. These proteins act by increasing the immune response of the host against microbial infections, thus overcoming their immunosuppressive mechanisms and offers an alternative to combat the increasing drug resistance. These approaches may also provide new insights on TLR biology and aid in the discovery of new targets glycosides useful in therapy.

### Conflict of interest statement

The Editor and Authors declare that while the authors and reviewer (Rafael E Silva) are affiliated with the same institution there has been no conflict of interest during the review and handling of this manuscript.
